# Latest from the WISE: Contributions to the Understanding of Ischemia and Heart Failure among Women with No Obstructive Coronary Arteries

**DOI:** 10.31083/j.rcm2403090

**Published:** 2023-03-15

**Authors:** Breanna Hansen, Michael D. Nelson, Eileen M. Handberg, Carl J. Pepine, C. Noel Bairey Merz, Janet Wei

**Affiliations:** ^1^Department of Medicine, Cedars-Sinai Medical Center, Los Angeles, CA 90048, USA; ^2^Department of Kinesiology, University of Texas at Arlington, Arlington, TX 76019, USA; ^3^Division of Cardiology, Department of Medicine, University of Florida, Gainesville, FL 32611, USA; ^4^Barbra Streisand Women’s Heart Center, Smidt Heart Institute, Cedars-Sinai Medical Center, Los Angeles, CA 90048, USA

**Keywords:** coronary microvascular dysfunction, women, ischemic heart disease, heart failure with preserved ejection fraction, cardiac magnetic resonance

## Abstract

Since 1996, the National Heart, Lung, and Blood Institute-sponsored Women’s Ischemia Syndrome Evaluation (WISE) has been investigating pathophysiological processes underlying ischemic heart disease in women and related outcomes. Recent findings have focused on women with signs and symptoms of ischemia and no obstructive coronary arteries (INOCA) and their elevated risk for heart failure with preserved ejection fraction (HFpEF). This review summarizes the latest WISE findings related to INOCA and pre-HFpEF characteristics, addressing our understanding of contributions from traditional vs nontraditional risk factors in women.

## 1. Introduction

At least 3–4 million American women and men are estimated to have ischemia with no obstructive coronary arteries (INOCA) [1, 2]. For the past 25 years, the National Heart, Lung and Blood Institute-sponsored Women’s Ischemia Syndrome Evaluation (WISE) has explored diverse aspects of ischemic heart disease (IHD) in women, with recent focus on INOCA [3, 4]. Women with INOCA are often dismissed from optimal medical therapy [5, 6, 7, 8] and are managed mainly for their traditional cardiovascular risk factors and not as a spectrum of ischemic heart disease [9]. This is problematic given that women with INOCA are at elevated risk of major adverse cardiovascular events, including a 10-fold increased rate of heart failure [10], confirmed to be mostly heart failure with preserved ejection fraction (HFpEF) [11]. A majority of women with suspected INOCA have coronary endothelial and coronary microvascular dysfunction (CMD), as measured by invasive coronary functional testing [12]. Intriguingly, there is growing evidence linking CMD with risk for HFpEF [13, 14]. For both INOCA and HFpEF, there is an urgent need for effective therapies [15], some of which may require tailoring specifically for women vs men [16, 17]. As described in our 2020 review [18], many knowledge gaps and questions remain in understanding at-risk INOCA phenotype, progression to HFpEF, and developing an evidence-base to support diagnostic, prognostic, and management guidelines. Recent WISE studies that fill in some of these research gaps and investigate contributions to ischemia and heart failure in women with no obstructive coronary arteries are summarized here.

## 2. Coronary Vascular Dysfunction in INOCA

**Fig. 1.**
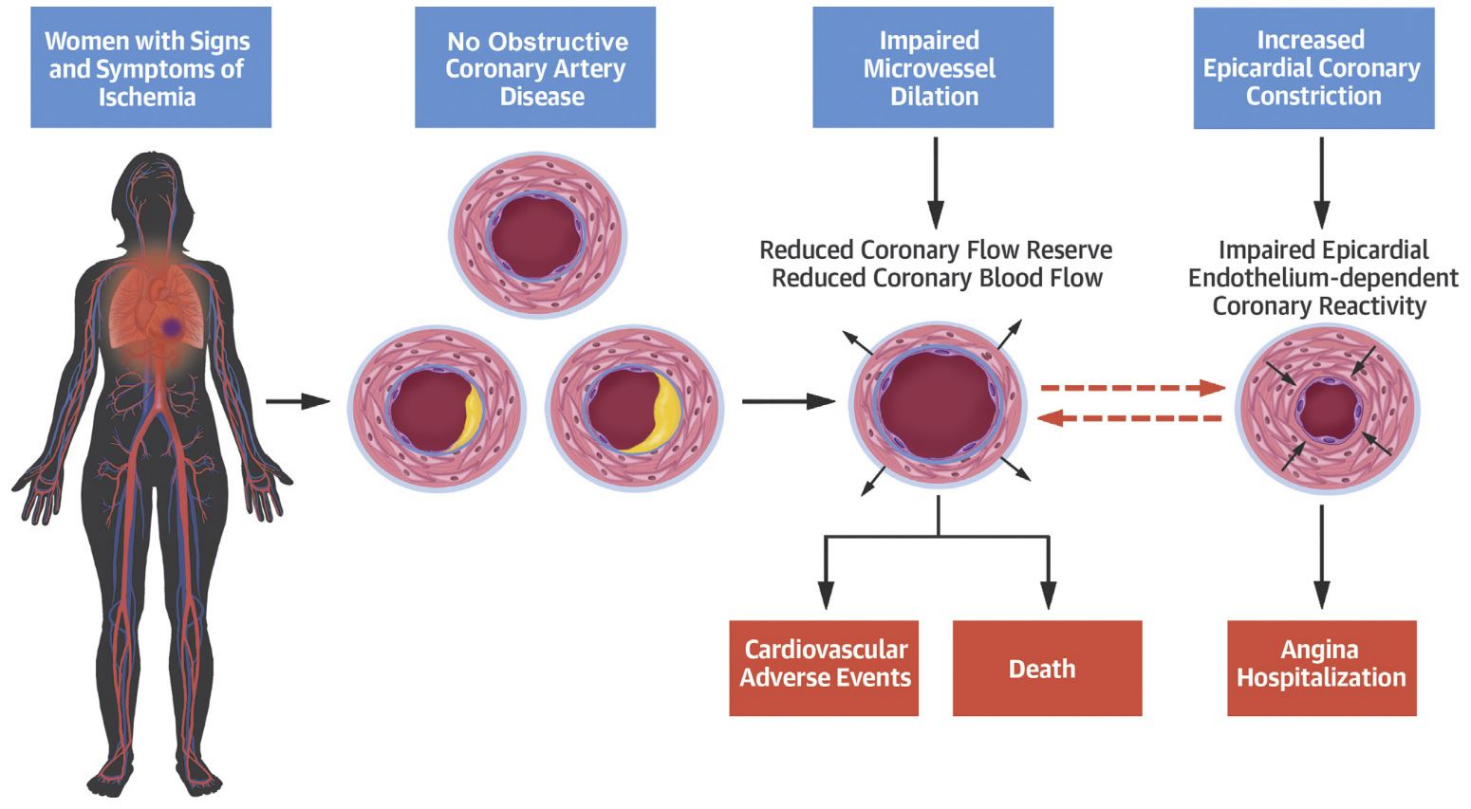
**Abnormal coronary microvascular and macrovascular function in women with INOCA**. In women with signs and symptoms of INOCA, invasive coronary function testing identifies abnormalities in coronary microvascular and macrovascular function that predict adverse cardiovascular outcomes. INOCA, ischemic with no obstructive coronary arteries (Reprinted with permission [20]).

Women with suspected INOCA often have coronary vasomotor dysfunction of the microvascular and/or macrovascular coronary arteries, which contribute to major adverse cardiovascular events including death, nonfatal myocardial infarction, nonfatal stroke, and hospitalization for heart failure and/or angina (Fig. 1) [19, 20]. The WISE previously reported that nearly half of women with suspected INOCA have impaired coronary flow reserve (CFR <2.5) and a majority have endothelial dysfunction [3, 12]. Many of these women in the WISE original cohort did not have hypertension, dyslipidemia, diabetes, or active smoking, consistent with prior findings that traditional risk factors do not fully explain coronary vascular dysfunction in women [21, 22]. Furthermore, commonly used cardiovascular disease risk scores (e.g., Framingham Risk Score, Reynolds Risk Score, Pooled Cohort Equations) fail to accurately predict cardiovascular outcome rates in women with INOCA, often underestimating risk [23].

Expert consensus documents and practice guidelines have recently highlighted the diagnosis of INOCA endotypes (CMD and coronary vasospasm) in patients with chest pain and no obstructive coronary artery disease (CAD) [24, 25]. The guidelines recommend assessing CMD using invasive coronary function testing, stress positron emission tomography with assessment of myocardial blood flow reserve (MBFR), or stress CMR with assessment of MBFR. While advances in invasive and noninvasive modalities have helped to increase diagnosis of INOCA over time, unfortunately angina hospitalization rates have continued at a relatively constant rate in women with INOCA [26]. The recent ACC/AHA chest pain guideline emphasized the underdiagnosis of women with chest pain and advised moving away from “atypical” versus “typical” chest pain and towards “cardiac” versus “non-cardiac” chest pain to reduce underdiagnosis of ischemic chest pain and improve IHD outcomes in women [25].

Women not uncommonly have impaired coronary flow reserve due to high resting coronary blood flow, which is associated with higher cardiovascular mortality risk compared to those with preserved coronary flow reserve [27]. Not well understood is the significance of higher resting coronary blood flow in women compared to men with INOCA, thought to be related to sex differences in autonomic nervous system function and estradiol receptor mediated vasodilation [19]. Studies from the WISE-Coronary Vascular Dysfunction Project observed those women with higher resting coronary blood flow had lower CFR and worse angina scores. This finding suggests that higher resting coronary flow may represent either disordered autoregulation or appropriate autoregulation in response to an increase in myocardial oxygen demand. Others have shown that elevated resting flow can be mediated by increased nitric oxide synthase activity [28] or by defective cardiomyocyte substrate utilization [29]. This high resting flow subtype may be a specific pathophysiologic contributor to CMD and/or its related symptom expression [30]. Furthermore, among WISE subjects with impaired CFR <2.32, prevalence of cardiovascular risk factors and coronary plaque burden were similar between those with high vs low resting coronary flow, but women with low resting coronary flow had higher LV end-diastolic filling pressure, lower LV ejection fraction and worse systolic and diastolic strain [31]. Thus, the combination of low resting coronary flow and impaired CFR may identify an INOCA phenotype at greatest risk for heart failure.

While not formally described as a distinct endotype of INOCA, myocardial bridging is a clinically relevant finding on coronary angiography, associated with atherosclerotic development in the segment proximal to the myocardial bridge [32], and predicts epicardial and microvascular endothelial dysfunction as measured during intracoronary acetylcholine infusion [33]. Myocardial bridging has recently been found to be an independent predictor of acetylcholine-induced epicardial or microvascular spasm and myocardial infarction in the setting of no obstructive coronary arteries (MINOCA) [34]. Compared to patients without myocardial bridge, those with myocardial bridge had a higher rate of major adverse cardiac events and worse angina-related quality of life [34]. Furthermore, the combination of myocardial bridge presence with acetylcholine-induced spasm was associated with a higher rate of recurrent angina compared with myocardial bridge presence without spasm [35]. Although myocardial bridging has been linked with left ventricular systolic dyssynchrony [36], relation to heart failure progression is unknown. Current WISE studies are gathering data about myocardial bridging and subsequent heart failure outcomes.

Given the heterogeneity of INOCA mechanisms, recent randomized trials have helped to guide INOCA therapy based on CMD vs vasospasm endotype [37, 38], and ongoing trials are testing novel therapies and clinical strategies [39, 40, 41, 42, 43, 44]. WISE investigators are leading a large prospective randomized open blinded end-point trial (clinicaltrials.gov ID: NCT03417388) to determine whether intensive medical therapy with high-intensity statin, angiotensin converting enzyme inhibitor (ACE-I) or receptor blockers (ARB) and aspirin will reduce cardiovascular outcomes compared to usual care in women with suspected INOCA [45].

## 3. Vascular Dysfunction: A Systemic Disease in INOCA?

Growing evidence suggest that coronary vascular dysfunction may be a manifestation of a systemic process in women with INOCA. A collaboration of WISE with the Microvascular Aging and Eicosanoids –Women’s Evaluation of Systemic aging Tenacity (MAE-WEST) Specialized Center of Research Excellence (SCORE) on Sex Differences is investigating the microvascular aging effects on brain, heart, and kidney function in patients with INOCA. The SCORE project will study sex-specific association of inflammatory eicosanoid mediators with microvascular aging physiology and the role of intensive medical treatment on inflammatory profiles and total microvascular disease burden [46]. Similarly, an ongoing ancillary WISE study will test the hypothesis that cerebral small vessel disease is directly related to microvascular disease burden by evaluating brain magnetic resonance imaging in women and men with CMD.

The WISE previously observed that mild chronic renal insufficiency is significantly associated with CMD in women with suspected INOCA and an independent predictor of cardiovascular mortality regardless of CAD severity in women [47, 48]. Recent evaluation of urine albumin-creatine ratio (UACR) among women with INOCA demonstrated that renal endothelial dysfunction was directly related to endothelial-dependent CMD, as measured by coronary blood flow change in response to intracoronary acetylcholine [49]. In multivariable regression modeling, UACR was the second strongest predictor of endothelial-dependent CMD after low-density lipoprotein (LDL)-cholesterol. These results suggested that endothelial-dependent CMD in women with suspected INOCA may be a manifestation of a systemic process and targeting microalbuminuria may have prognostic and treatment implications for women with INOCA.

If microvascular dysfunction is a systemic process in patients with INOCA, then further studies are needed to determine if multiorgan microvascular dysfunction contributes to HFpEF progression in INOCA patients. Indeed, HFpEF is known to be a multiorgan systemic disorder associated with multiorgan reserve dysfunction [15]. However, given the complexity of risk factors and pathophysiologies in HFpEF, it remains unclear whether microvascular dysfunction

## 4. Cardiac Autonomic Function and Ischemia

The cardiac autonomic nervous system is an important regulator of coronary blood flow and encompasses parasympathetic and sympathetic factors [50]. While the cardiac autonomic nervous dysfunction may explain a high prevalence of silent ischemia in the setting of obstructive CAD or diabetes mellitus [51, 52], cardiac autonomic dysfunction in the setting of INOCA remains incompletely understood. In a WISE-related study, 36 women with suspected INOCA in the absence of obstructive CAD were found to have a high prevalence of silent ischemia on 24-hour 12-lead ambulatory ECG monitoring, with the majority occurring in the absence of sinus tachycardia [53]. A subset of these women also ${\mathrm{underwent}}^{123}$I- *meta* ${\mathrm{-iodobenzylguanidine (}}^{123}$I- *m* IBG) scans to investigate the relationship between cardiac sympathetic activity and INOCA. Retention of ${\mathrm{the}}^{123}$I- *m* IBG radionucleotide is a reflection of neuronal integrity and low myocardial uptake has previously been found to predict adverse outcomes heart failure [54, 55]. Greater than one-fourth of women with suspected INOCA demonstrated low ${\mathrm{late}}^{123}$I- *m* IBG uptake (as defined as heart-mediastinal ratio <1.6) and high washout ratio (>27%) compared to reference women, suggestive of cardiac sympathetic dysregulation [56]. The authors noted an inverse relationship between heart-mediastinal ratio and endothelial function and hypothesized that cardiac sympathetic dysregulation may be due to chronic recurrent ischemia in the setting of suspected INOCA. Further investigation is needed to determine whether ${\mathrm{low}}^{123}$I- *m* IBG uptake may identify INOCA patients at higher risk for heart failure.

One method to stimulate the sympathetic nervous system is cold pressor testing, which increases myocardial blood flow by dilation of coronary arterioles. However, cold pressor testing paradoxically causes vasoconstriction in coronary segments with CAD or endothelial dysfunction [57]. Among 107 WISE women with suspected INOCA and 20 asymptomatic age-matched reference women who underwent stress-rest cardiac magnetic resonance (CMR) perfusion with cold pressor testing, myocardial perfusion reserve to cold pressor testing was greater among women with INOCA compared to reference women, even in those with coronary endothelial dysfunction [58]. The authors concluded that the ability for cold pressor testing to induce a higher myocardial perfusion response among INOCA women compared to reference women reflects underlying differences in sympathetic autonomic resting tone and response, possibly due to heightened sympathetic innervation or sympathetic nervous system hyperresponsiveness. In this study, the INOCA women with endothelial dysfunction had lower resting ejection fraction and higher mass-volume ratio, indices of left ventricular (LV) dysfunction and remodeling. Thus, further exploration of the link between cardiac sympathetic activity and INOCA in relation to development of heart failure is needed.

## 5. Psychologic Health and Mental Stress in INOCA

Depression and anxiety are established risk factors for IHD and adverse cardiovascular outcomes, particularly in women [59]. Depression and anxiety were related to angina frequency and IHD outcomes in WISE studies [60, 61]. Furthermore, somatic (e.g., fatigue and sleep impairment) but not cognitive (e.g., loss of interest and pessimism) depressive symptoms were found to predict an increased risk of obstructive CAD, cardiovascular-related mortality and events [62].

The WISE subsequently studied 901 women with INOCA and found that home/work stress or financial stress was associated with greater cardiac symptoms, functional impairment, and CAD risk factors [63]. Mechanisms through which different forms of psychologic stress could affect CAD risk are broad, from direct physiologic disruptions in the hypothalamic-pituitary adrenal axis, brain neuronal death, autoimmune inflammation, and telomere shortening, to indirect disruptions related to negative behavioral responses and predisposition to the development of CAD risk factors. Indeed, mental stress-induced myocardial ischemia is frequent in patients with CAD, including young women with recent myocardial infarction [64]. In a WISE related study, women with suspected INOCA who underwent peripheral artery tonometry (PAT) had more peripheral vasoconstriction to mental-stress compared to age-matched reference women [65], possibly related to the autonomic dysregulation as the stress PAT ratio did not correlate with any of the coronary function measures defined during invasive coronary function testing. Interventions that modulate autonomic vasoconstrictive responses should be tested in INOCA women with high levels of psychologic stress.

## 6. Adverse Pregnancy Outcomes in Women with INOCA

Adverse pregnancy outcomes (such as hypertensive disorders of pregnancy (HDP), gestational diabetes, and intrauterine growth restriction) are associated with increased risk of IHD in women [66], but the underlying mechanisms remain unclear. In a WISE study of 184 women with suspected INOCA who underwent invasive coronary function testing, history of adverse pregnancy outcome was associated with lower coronary flow reserve (CFR) indicative of CMD [67]. The authors found that addition of hypertension history strengthened the association between adverse pregnancy outcomes and CFR, suggesting that hypertension may mediate the association between adverse pregnancy outcomes and limited CFR [67]. Indeed, the WISE observed that among women with INOCA, a history of HDP was associated with 3.2-fold increased odds of chronic hypertension [68]. Women with a history of both HDP and chronic hypertension had higher LV mass than those with HDP alone, highlighting the higher risk for cardiac remodeling in women with concomitant history of HDP and chronic hypertension. Since HDP has been associated with increased risk of heart failure and this association was largely independent of CAD [69], further work is needed to understand whether INOCA contributes to this risk.

## 7. Pre-HFpEF Characteristics in Women with INOCA

HFpEF is characterized by LV myocardial dysfunction, with impaired LV diastolic function and subclinical systolic dysfunction [15]. Impaired CFR is present in the majority of patients with HFpEF, thought to be related to diffuse microvascular inflammation, microvascular dysfunction, microvascular rarefaction, along with left ventricular stiffness [14, 15]. Thus, WISE investigators hypothesize that CMD in women with suspected INOCA may play a critical role in a “pre-HFpEF” state [70].

Left ventricular end-diastolic pressure is elevated in ~40% in women with INOCA, and LV diastolic function (measured noninvasively through imaging) is often impaired compared to age-matched reference women [71, 72]. Recent WISE studies have provided further insight into LV diastolic and systolic strain of women with suspected INOCA [73, 74]. In a case-control comparison of 128 women with CMD (as defined as abnormal invasive CFR, coronary endothelial dysfunction, or myocardial perfusion reserve index [MPRI] <2.0) and 43 healthy age-matched reference women, both peak systolic and diastolic circumferential strain and radial strain were lower in CMD cases compared to reference controls, despite similar and preserved ejection fraction [73]. The CMD group had lower MPRI and greater prevalence of cardiovascular risk factors than the reference controls [75, 76]. These results demonstrated that both diastolic and systolic dysfunction are present in CMD women, but mechanisms contributing to the LV dysfunction (ischemia vs risk factors) remained poorly understood.

Since INOCA is a heterogenous condition and only a subset of patients progresses to HFpEF, WISE investigators strive to identify INOCA subgroups at risk for HFpEF. To test the hypothesis that non-obstructive ischemia contributes to worse LV dysfunction, WISE analyzed 317 symptomatic women with suspected INOCA who underwent CMR and found that women with impaired MPRI <1.84 (mean MPRI 1.49 ± 0.25) had higher circumferential strain and LV ejection fraction and no difference in diastolic strain rate than those with MPRI ≥1.84 (mean MPRI 2.28 ± 0.35 [74]. Although these findings were contrary to the original hypothesis, studies have indicated that compensatory increased circumferential shortening occurs in the transition from pre-clinical dysfunction to HFpEF, suggesting that the INOCA subgroup with low MPRI may have pre-clinical HFpEF [77].

Recent WISE CMR studies have evaluated other cardiac structural and functional abnormalities in INOCA women. Among 65 women with suspected INOCA and 12 healthy reference women, aortic pulse wave velocity (aPWV), elevated left ventricular mass index, and lower extracellular volume were independent predictors of diastolic dysfunction, as represented by decreased early diastolic circumferential strain rate [78]. Systolic blood pressure was not a significant independent predictor of diastolic dysfunction, and MPRI did not appear to differentiate between normal and abnormal diastolic function in this cohort. These findings suggest that aortic stiffness, and associated ventricular-arterial uncoupling, contribute to myocardial hypertrophy and LV diastolic dysfunction in women with INOCA. Increased left atrial stiffness may also accompany ventricular dysfunction in women with INOCA [79]. Future studies are needed to determine if lowering aortic and left atrial stiffness can prevent development of HFpEF in this population.

Plasma and serum biomarkers may further elucidate mechanism of LV dysfunction in women with INOCA. WISE found ultra-high-sensitivity cardiac troponin I (u-hsc-TnI) levels were significantly elevated in women with coronary endothelial dysfunction diagnosed by invasive coronary function testing [80]. Subsequent WISE analysis of 327 women with INOCA determined that higher u-hscTnI levels were associated with higher LV mass index and strain measures of LV diastolic and LV systolic dysfunction [81], expanding prior reports linking hs-cTnI with echocardiographic measures of LV diastolic dysfunction and heart failure hospitalizations in patients with suspected INOCA [13]. These data suggest that cardiomyocyte injury is the underlying mechanism of LV systolic and diastolic dysfunction in patients with INOCA, supporting the link between CMD-related ischemia, cardiomyocyte injury, LV diastolic dysfunction, and subsequent heart failure hospitalization. Oxidative stress, as measured by elevated levels of plasma cystine or lower levels of glutathione, may also play a role in diastolic dysfunction in women with INOCA [82] and has previously been shown to be an independent predictor [83].

A model combining traditional, novel, and sex-specific risk factors may also help identify high risk INOCA women at risk for HFpEF development. In a retrospective analysis of 493 WISE women with no obstructive CAD and no prior history of heart failure, diabetes mellitus and tobacco use were associated with heart failure hospitalization at a median follow-up of 6-years [84]. In a multivariate analysis adjusting for traditional heart failure predictors (age, diabetes, hypertension, tobacco use, statin use), WISE identified the following novel predictors of heart failure hospitalization: higher resting heart rate, parity, interleukin-6 levels, lower CFR and poor functional status. Although N-terminal pro-B-type natriuretic peptide (NT-proBNP) has been established as a predictor of progression in HFpEF, NT-proBNP may be normal in patients with HFpEF and influenced by presence of atrial fibrillation, renal dysfunction and obesity [85]. In a WISE study of 208 women with INOCA who underwent invasive coronary function testing, those with elevated >400 pg/mL with NT-proBNP had lower CFR, but NT-proBNP did not correlate with LVEDP and was not independently associated with CFR [86], suggesting that NT-proBNP levels at rest may not be useful in risk assessment of INOCA women unless they are significantly elevated >400 pg/mL.

A novel plasma biomarker of cardiomyocyte calcium handling protein, cardiac bridge interrogator 1 (cBIN-1), was recently found to be elevated in women with INOCA, reflecting cardiomyocyte tubule dysfunction [87]. Furthermore, higher cBIN1 score was associated with coronary endothelial dysfunction in these women. This finding adds to existing literature that indicate that coronary endothelial dysfunction may eventually lead to changes within the myocytes resulting in fibrosis, ventricular remodeling and heart failure [88].

Could improvement in INOCA alter the risk for HFpEF development? Utilizing baseline and one-year follow-up CMR data of women with suspected INOCA who were clinically treated with cardiac medication, WISE observed concurrent temporal trends toward improvement in angina, myocardial perfusion, LV morphology and function, and blood pressure [89]. In addition, abnormalities in LV morphology and diastolic function at baseline (e.g., higher LV end-diastolic volume and time to peak filling rate) were predictive of clinically significant improvement in angina at follow-up, whereas history of hypertension was associated with decreased odds of angina improvement. These results support the interrelationship between angina and LV morphology and function. Future studies are needed to determine the mechanisms and treatments responsible for the observed improvements.

In summary, mechanisms linking INOCA and HFpEF appear to be coronary non-endothelial dysfunction and endothelial-dependent dysfunction and cardiac myocyte injury. Endothelial dysfunction is associated with lower resting ejection fractions and higher mass-volume ratios with indices of LV dysfunction and remodeling. INOCA women with worse coronary endothelial function had higher u-hscTnl levels which were in turn associated with impaired LV diastolic and systolic dysfunction. Additionally, women with INOCA who had impaired CFR (<2.5) had higher LV end-diastolic filling pressure, lower LV ejection fraction and worse systolic and diastolic strain.

**Fig. 2.**
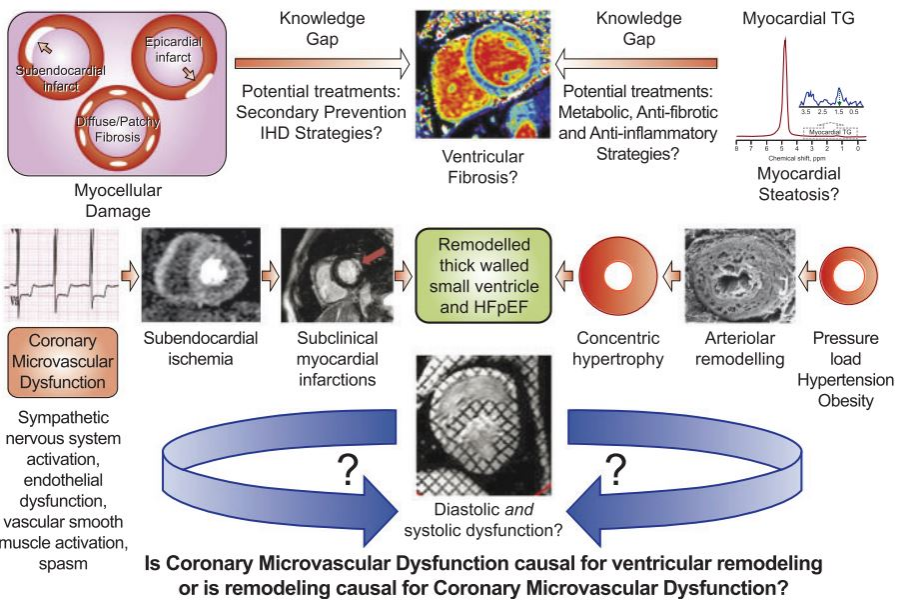
**The Chicken or the Egg**? Studies from the WISE are evaluating mechanisms contributing to higher risk of HFpEF in patients with INOCA. Prior WISE studies suggest that myocardial cellular damage and myocardial steatosis may contribute to myocardial remodeling and dysfunction in women with INOCA, but significant knowledge gaps exist regarding potential therapies. While traditional cardiovascular risk factors such as hypertension and obesity are common in patients with INOCA and known to contribute to myocardial remodeling, emerging evidence suggest that CMD is associated with subclinical functional and structural abnormalities. However, causal mechanistic links and the sequence of causality remain unknown. Does CMD lead to left ventricular remodeling and dysfunction or is ventricular modeling and dysfunction causal for CMD? Future work is needed to fill key knowledge gaps. CMD, coronary microvascular dysfunction; IHD, ischemic heart disease; INOCA, ischemia with no obstructive coronary arteries; HFpEF, heart failure with preserved ejection fraction; TG, triglycerides; WISE, Women’s Ischemia Syndrome Evaluation (Reprinted with permission [90]).

While WISE investigators and others have suggested that INOCA and HFpEF appear associated, causal mechanistic links and the sequence of causality remain unknown (Fig. 2) [90]. Further investigations to the mechanisms of myocardial remodeling and dysfunction are underway. Currently, two cohorts are being enrolled in WISE studies to evaluate mechanistic links between suspected INOCA and HFpEF: (1) women and men with HFpEF (WISE-HFpEF) (NCT02582021), (2) women and men with CMD undergoing invasive coronary function testing (WISE - Pre-HFpEF) (NCT03876223). A third WISE-related study is evaluating the potential link between CMD-related ischemia, myocardial steatosis, and pre-HFpEF traits [91].

## 8. Conclusions

A comprehensive review of coronary arterial function and disease in women with INOCA recently outlined research gaps and expanded research recommendations to improve the understanding, diagnosis, and management of INOCA in women [19]. In 2020, the National Heart, Lung, and Blood Institute published research priorities for HFpEF and highlighted CMD as an important “endophenotype” of INOCA for identifying pathobiological mechanisms and developing targeted interventional trials in HFpEF [15]. This current review of WISE studies demonstrates that many knowledge gaps remain in the mechanistic link between suspected INOCA and HFpEF in patients with INOCA. Ongoing and future WISE investigations will aim to fill in these gaps in both women and men.
